# Analysis of a taurine-dependent promoter in *Sinorhizobium meliloti* that offers tight modulation of gene expression

**DOI:** 10.1186/s12866-014-0295-2

**Published:** 2014-11-25

**Authors:** Mina Mostafavi, Jainee Christa Lewis, Tanisha Saini, Julian Albert Bustamante, Ivan Thomas Gao, Tuyet Thi Tran, Sean Nicholas King, Zhenzhong Huang, Joseph C Chen

**Affiliations:** Department of Biology, San Francisco State University, San Francisco, CA 94132 USA

**Keywords:** *Alphaproteobacteria*, Rhizobia, *Sphingomonadaceae*, Transcriptional regulator, Beta-glucuronidase, mCherry, Taurine metabolism, Essential genes, Twin arginine translocation

## Abstract

**Background:**

Genetic models have been developed in divergent branches of the class *Alphaproteobacteria* to help answer a wide spectrum of questions regarding bacterial physiology. For example, *Sinorhizobium meliloti* serves as a useful representative for investigating rhizobia-plant symbiosis and nitrogen fixation, *Caulobacter crescentus* for studying cell cycle regulation and organelle biogenesis, and *Zymomonas mobilis* for assessing the potentials of metabolic engineering and biofuel production. A tightly regulated promoter that enables titratable expression of a cloned gene in these different models is highly desirable, as it can facilitate observation of phenotypes that would otherwise be obfuscated by leaky expression.

**Results:**

We compared the functionality of four promoter regions in *S. meliloti* (P_*araA*_, P_*tauA*_, P_*rhaR*_, and P_*melA*_) by constructing strains carrying fusions to the *uidA* reporter in their genomes and measuring beta-glucuronidase activities when they were induced by arabinose, taurine, rhamnose, or melibiose. P_*tauA*_ was chosen for further study because it, and, to a lesser extent, P_*melA*_, exhibited characteristics suitable for efficient modulation of gene expression. The levels of expression from P_*tauA*_ depended on the concentrations of taurine, in both complex and defined media, in *S. meliloti* as well as *C. crescentus* and *Z. mobilis*. Moreover, our analysis indicated that TauR, TauC, and TauY are each necessary for taurine catabolism and substantiated their designated roles as a transcriptional activator, the permease component of an ABC transporter, and a major subunit of the taurine dehydrogenase, respectively. Finally, we demonstrated that P_*tauA*_ can be used to deplete essential cellular factors in *S. meliloti*, such as the PleC histidine kinase and TatB, a component of the twin-arginine transport machinery.

**Conclusions:**

The P_*tauA*_ promoter of *S. meliloti* can control gene expression with a relatively inexpensive and permeable inducer, taurine, in diverse alpha-proteobacteria. Regulated expression of the same gene in different hosts can be achieved by placing both *tauR* and P_*tauA*_ on appropriate vectors, thus facilitating inspection of conservation of gene function across species.

**Electronic supplementary material:**

The online version of this article (doi:10.1186/s12866-014-0295-2) contains supplementary material, which is available to authorized users.

## Background

The class *Alphaproteobacteria* encompasses a multitude of noteworthy taxa with diverse physiologies. Members of the group range from human and livestock pathogens, such as *Rickettsia* and *Brucella*, to rhizobial species, such as *Sinorhizobium meliloti* and *Agrobacterium tumefaciens* (also known as *Ensifer meliloti* and *Rhizobium radiobacter*, respectively), that benefit or damage agricultural crops [1]. The possible progenitor of present-day mitochondria, and several highly abundant marine groups, including *Roseobacter*, SAR116, and SAR11, also belong to this class [2]. In-depth molecular studies of various representatives have led to significant insights regarding cell cycle progression [3], organelle biogenesis [4], metabolic pathways [5], microbe-host interactions [6,7], and potential for biofuel production [8].

The availability of genetic tools in readily culturable bacteria facilitates the investigation of their physiologies. In particular, an inducible promoter that allows tight titration of gene expression enables functional analysis of cellular factors that are toxic at low levels or essential for viability. For example, investigators working with *Escherichia coli*, a gamma-proteobacterium, possess an array of such tools at their disposal: some commonly used ones are the arabinose-inducible P_BAD_ promoter [9,10], the rhamnose-inducible P_*rhaB*_ promoter [11], and various derivatives of the original *lac* and *tet* promoters [12,13]. Within the alpha-proteobacteria group, *Caulobacter crescentus* is perhaps one of the representative members most amenable to genetic manipulation, with two well characterized, regulatable promoters, inducible by xylose or vanillate [14-16]. Identification of these control elements has helped *C. crescentus* emerge as a prominent model for study of bacterial cell biology [17].

In contrast, the agriculturally important alpha-proteobacterium *S. meliloti*, which forms mutualistic symbiosis with legume plants, lacks a selection of such fully developed induction systems. Following reciprocal exchange of chemical signals between a suitable plant host and the endosymbiont, *S. meliloti* infects and colonizes nodules that develop along the plant root; the bacteria enter membrane-bound compartments within cortical cells and differentiate into bacteroids, capable of converting atmospheric dinitrogen to ammonia [6,18]. The ability of *S. meliloti* to fix nitrogen for its hosts, especially alfalfa, improves crop yield, reduces expensive reliance on synthetic fertilizers, and enriches soil fertility in an ecologically sound manner [19,20]. Better tools for manipulating the bacterium would facilitate investigation of this model rhizobial-plant symbiosis. Our goal is to identify expression systems that can be modulated and efficiently shut off, thus permitting characterization of phenotypes that would otherwise be obscured by leaky expression. We are particularly interested in a system that is functional and easily transferrable among different alpha-proteobacteria. Derivatives of the *E. coli lac* promoter, inducible by isopropyl-beta-D-thiogalactopyranoside (IPTG), appear to serve this purpose [21,22]. Nevertheless, additional candidates that would enlarge the repertoire of genetic tools deserve further examination.

**Figure 1:**
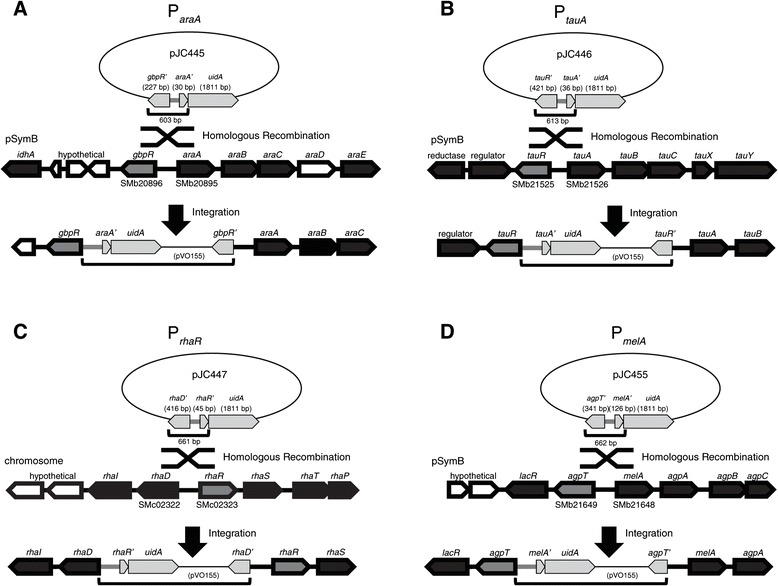
**Schematics depicting integration of plasmids into the** ***S. meliloti*** **genome to generate transcriptional fusions.** Integration of **(A)** pJC445, **(B)** pJC446, **(C)** pJC447, and **(D)** pJC455 resulted in fusions of the *uidA* reporter to promoter regions upstream of *araA* (SMb20895), *tauA* (SMb21526), *rhaR* (SMc02323), and *melA* (SMb21648), respectively. The sizes of the regions that enabled homologous recombination are shown below each plasmid. *gbpR*, *tauR*, *rhaR*, and *agpT* represent potential or confirmed regulators of genes involved in arabinose (*ara*), taurine (*tau*), rhamnose (*rha*), or melibiose/alpha-galactoside (*mel*/*agp*) metabolism. White pentagonal blocks indicate genes without assigned function.

We chose to compare four potential promoter regions--P_*araA*_, P_*tauA*_, P_*rhaR*_, and P_*melA*_--each with varying degrees of prior characterization. We selected these candidates because (1) their respective inducers are relatively affordable; (2) previous studies suggested potentials for modulating gene expression by at least one order of magnitude; and (3) genomic integrations of transcriptional fusions to the promoters can be achieved easily without disrupting genes involved in the metabolic pathways under investigation. The P_*araA*_ promoter region is located on the pSymB megaplasmid, upstream of the *araABCDEF* operon (Figure 1A), which was shown to be required for arabinose catabolism and inducible by arabinose more than 100-fold in defined medium [23]. However, expression may be subject to a certain degree of catabolite repression and inducer exclusion [23,24]. P_*tauA*_, P_*rhaR*_, and P_*melA*_ expression were tested in a large-scale screen for induction profiles of solute transporters and found to increase at least 100-, 6-, and 60-fold when cells were exposed to taurine (2-aminoethanesulfonate), rhamnose, or melibiose, respectively [25]. Located on pSymB and upstream of genes thought to be involved in taurine assimilation (Figure 1B) [26,27], the P_*tauA*_ promoter region has allowed controlled expression of reporter genes in rhizobial and *Rhodobacter* species [28] and of the site-specific recombinase Cre in *S. meliloti* [29]. The P_*rhaR*_ promoter region is located upstream of *rhaR*, which encodes a transcriptional regulator, and between chromosomal genes involved in rhamnose metabolism (Figure 1C) [30]. The P_*melA*_ promoter is located between *agpT* and *melA* on the pSymB megaplasmid (Figure 1D); *melA* and downstream genes are required for assimilation of alpha-galactosides, such as raffinose and melibiose, while AgpT is an AraC-like transcriptional activator, required for induction of the P_*melA*_ promoter [31,32]. P_*melA*_ has been used successfully in physiological studies of *S. meliloti* [33] and as a biosensor for environmental galactosides [34], but catabolite repression and inducer exclusion by succinate and other preferred compounds can also affect its induction [35,36].

We used transcriptional fusions to the genomic loci of these four promoters to assess their induction profiles in both complex and defined media. Our results suggested that P_*tauA*_ and P_*melA*_ offered both tighter regulation and wider ranges of expression compared to the other promoters. Further characterization of the P_*tauA*_ promoter indicated that the surrounding *tau* genes are required for taurine metabolism and affect expression from the promoter. We demonstrate the utility of the promoter by showing that P_*tauA*_ allows modulated expression of reporter genes in two other model alpha-proteobacteria, *C. crescentus* and *Zymomonas mobilis*. Moreover, we verified that P_*tauA*_ enables tight shut-off and depletion of essential genes in *S. meliloti*. Thus, this study establishes the feasibility of using P_*tauA*_ as a regulatory switch in diverse alpha-proteobacteria.

## Results and discussion

### Comparison of promoter regions in complex and defined media

To identify a promoter region that allows controlled expression of desired genes, we compared the levels of inducible expression from four different potential promoters: P_*araA*_, P_*tauA*_, P_*rhaR*_, and P_*melA*_. These regions were chosen according to the criteria described above after reviewing previously published studies for candidates. Each promoter region was transcriptionally fused to the *uidA* gene [encoding the beta-glucuronidase (GUS) reporter enzyme] on a suicide plasmid, which was subsequently integrated into the *S. meliloti* genome via homologous recombination (Figure 1). We designed the constructs to minimize disruption of the target genomic regions, such that the surrounding genes and their regulation remained intact, by integrating between divergently transcribed genes. Strains carrying the reporter constructs were grown in complex (PYE, LB) or defined (M9, M9 + CAA) media for three hours to mid-log phase, with or without appropriate inducers: arabinose for P_*araA*_, taurine for P_*tauA*_, rhamnose for P_*rhaR*_, and melibiose for P_*melA*_. For comparison in M9 minimal medium, strains were first grown in M9 supplemented with glucose (M9G), washed with M9, and then grown for three hours in M9G or M9 plus inducer as the sole carbon source. Samples were subsequently harvested for measurement of GUS activities.

**Figure 2:**
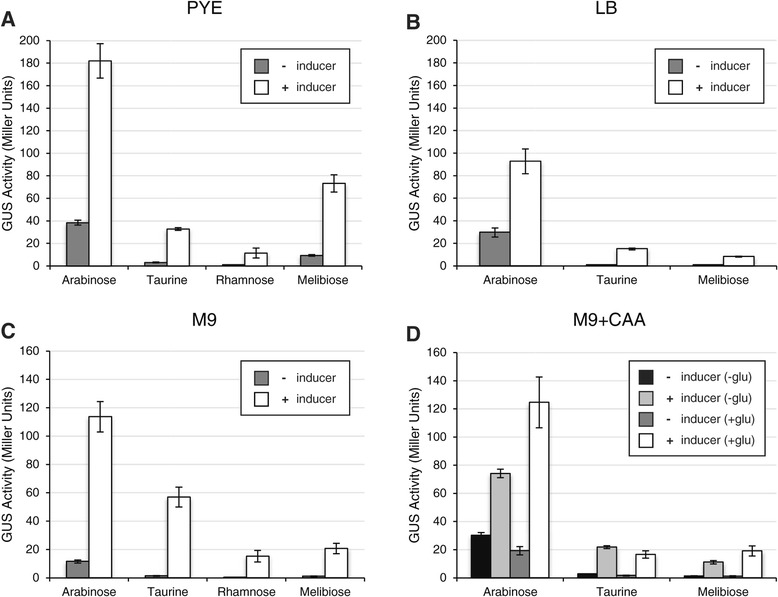
**Comparison of promoter strengths in different culture media.** Strains (JOE3273, JOE3275, JOE3277, and JOE3334) carrying transcriptional fusions of *uidA* to arabinose-, taurine-, rhamnose-, and melibiose-dependent promoter regions were grown in **(A)** PYE, **(B)** LB, **(C)** M9, or **(D)** M9 plus casamino acids (M9 + CAA) liquid media for three hours, to mid-log phase, in the presence or absence of appropriate inducers. Cultures were then harvested to measure the levels of beta-glucuronidase (GUS) expression. For M9 + CAA, strains were either first grown in M9 + CAA without glucose (−glu) and then diluted into the same medium with or without inducer; or they were first grown in M9 + CAA supplemented with 0.2% glucose (+glu) and diluted into M9 + CAA supplemented with the inducer [+ inducer (+glu)] or glucose [− inducer (+glu)]. Error bars indicate the standard errors of the mean GUS activities. See Methods for further details.

All strains exhibited higher levels of reporter gene expression in the presence of their respective inducers (Figure 2). For the P_*araA*_ promoter region, the maximal change occurred in M9 minimal medium, with a 10-fold increase in expression. Much larger changes in expression were reported in a previous study, possibly for a couple of reasons: (1) the induction time was longer; and (2) the reporter gene was inserted into *araF*, which is involved in arabinose catabolism [23]. Compared to other promoters, P_*araA*_ showed the highest level of induced expression in all rich and defined media tested: PYE, LB, M9, and M9 plus casamino acids (M9 + CAA). However, P_*araA*_ also showed relatively high levels of basal expression in the absence of arabinose. Basal expression was lowest in M9G (12 Miller units; Figure 2C), but it was still significantly higher than the basal expression of other promoters. Thus, while the P_*araA*_ promoter region is not suitable for tight regulation, it may serve as a useful tool for overexpression of desired genes.

The P_*rhaR*_ promoter demonstrated obvious elevation in expression when induced with rhamnose, increasing 10-fold (from 1.1 to 11 Miller units) in PYE complex medium and 38-fold (from 0.4 to 15.2 Miller units) in M9 minimal medium. However, P_*rhaR*_ also showed the lowest levels of expression, whether induced or uninduced, when compared to other promoters under similar conditions. We only measured expression from P_*rhaR*_ in PYE and M9 and did not test it in LB or M9 + CAA because other promoter regions all exhibited lower expression in LB compared to PYE and in M9 + CAA compared to M9. The results in PYE and M9 media had already suggested that the *rhaR* promoter region would not permit regulating genes that require high levels of expression.

The remaining two promoter regions, P_*tauA*_ and P_*melA*_, showed significant increases in GUS activities when induced. For P_*tauA*_, the increase ranged from 8-fold in M9 + CAA to 41-fold in M9, while for P_*melA*_ the increase ranged from 8-fold in PYE to 19-fold in M9. Both demonstrated low levels of basal expression and robust expression when induced. The notable exception occurred for P_*melA*_ in PYE, which exhibited higher levels of basal expression, suggesting leakiness in that particular medium (Figure 2A). Nevertheless, both P_*tauA*_ and P_*melA*_ emerged as useful candidates for modulating expression of desired genes.

**Figure 3:**
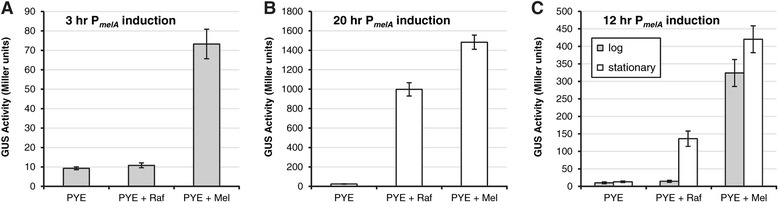
**Induction of P**_***melA***_ **promoter with melibiose (Mel) or raffinose (Raf).** Strain JOE3334, which carries the P_*melA*_-*uidA* reporter fusion, was grown in PYE rich medium and induced for **(A)** 3 hours, to mid-log phase; **(B)** 20 hours, to stationary phase; or **(C)** 12 hours, to mid-log or stationary phase. Error bars represent standard errors of the mean GUS activities. See Methods for further details.

In addition to melibiose (6-O-α-D-galactopyranosyl-D-glucose), we had also tested raffinose [O-α-D-galactopyranosyl-(1 → 6)-α-D-glucopyranosyl β-D-fructofuranoside] as a potential inducer for the P_*melA*_ promoter region. Unlike melibiose, raffinose led to very little change in reporter gene expression during the three-hour induction period in all media tested (Figure 3A; data not shown). Because this result contrasted with previous reports of raffinose as an inducer of *melA* and *agpA* expression [32,34], we examined induction of P_*melA*_ more closely in PYE. Induction occurred when the reporter strain carrying P_*melA*_*-uidA* was grown with raffinose or melibiose for 20 hours to stationary phase, with melibiose leading to stronger induction (Figure 3B). When the strain was grown with raffinose or melibiose for 12 hours, raffinose induced GUS expression if the culture reached stationary phase but not if the culture was maintained in log phase, whereas melibiose induced expression in either case (Figure 3C). Catabolic repression has been shown to influence induction of P_*melA*_ by raffinose [35,37], but melibiose seems to bypass this regulation in PYE rich medium. Therefore, raffinose appears to act as an inducer of the P_*melA*_ promoter in complex medium only if the culture reaches stationary phase, while melibiose is a more versatile inducer.

**Figure 4:**
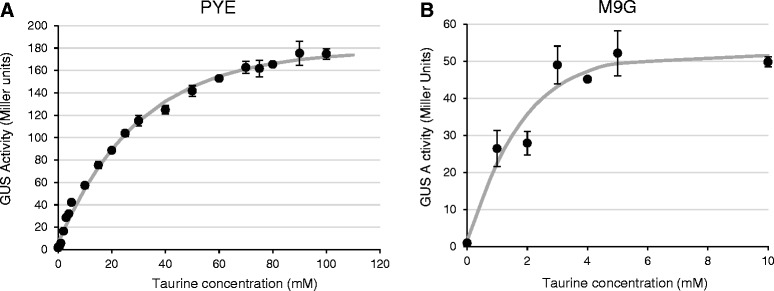
**Dose-dependent expression of the genomic P**_***tauA***_ **-** ***uidA*** **reporter fusion.** Strain JOE3275 was grown for 3 hours, to mid-log phase, in **(A)** PYE rich medium or **(B)** M9 plus 0.2% glucose (M9G) defined medium containing increasing concentrations of taurine and assayed for beta-glucuronidase (GUS) activity. (In contrast to the experiment described in Figure 2C, in which M9 contained taurine as the sole carbon source during induction, the M9 media in this experiment contained both glucose and taurine during induction.) Error bars indicate the standard errors of the mean GUS activities.

Because P_*tauA*_ exhibited low levels of basal activity and a potentially more dynamic range of expression compared to other promoter regions, we characterized it further. The reporter strain carrying the genomic P_*tauA*_*-uidA* fusion was used to monitor expression over a range of taurine concentrations, following a three-hour induction period, in both PYE (Figure 4A) and M9G (Figure 4B). The levels of expression exhibited clear dosage-dependent responses in both media. In PYE, the promoter approached maximal expression at 90 to100 mM taurine. (We did not use concentrations above 100 mM because adding higher levels of taurine to the medium became impractical, as its aqueous solubility is 500 mM.) Expression increased from 1.5 to 175 Miller units, a 117-fold change. In M9G medium, expression became saturated by 5 mM taurine. The range of expression was smaller, from an average of 1.0 to 52 Miller units, a 52-fold change. These results indicated that the P_*tauA*_ promoter exhibits a wide range of expression in both defined and complex media.

### Effects of *tauR*, *tauC*, and *tauY* on P_*tauA*_ expression

Next, we examined the effects of representative genes in the *tau* region (*tauR*, *tauC*, and *tauY*) on P_*tauA*_ expression. *tauR* (SMb21525) encodes a transcriptional regulator of the GntR family, *tauC* (SMb21528) encodes the permease component of a probable taurine ABC transporter, and *tauY* (SMb21529) encodes the large subunit of the putative taurine dehydrogenase [26,38]. Genetic analyses suggested that orthologs of the TauABC transporter in *E. coli* and *Rhodobacter capsulatus* participate in taurine utilization [39-41]. Also, previous studies indicated that the TauR ortholog in *R. capsulatus* (49% identity, 62% similarity, 0% gap [42]) activates expression of genes necessary for taurine dissimilation [43], and *S. meliloti* P_*tauA*_ requires its cognate TauR for expression in *Rhizobium leguminosarum* [28]. We expected similar behaviors in *S. meliloti*.

**Figure 5:**
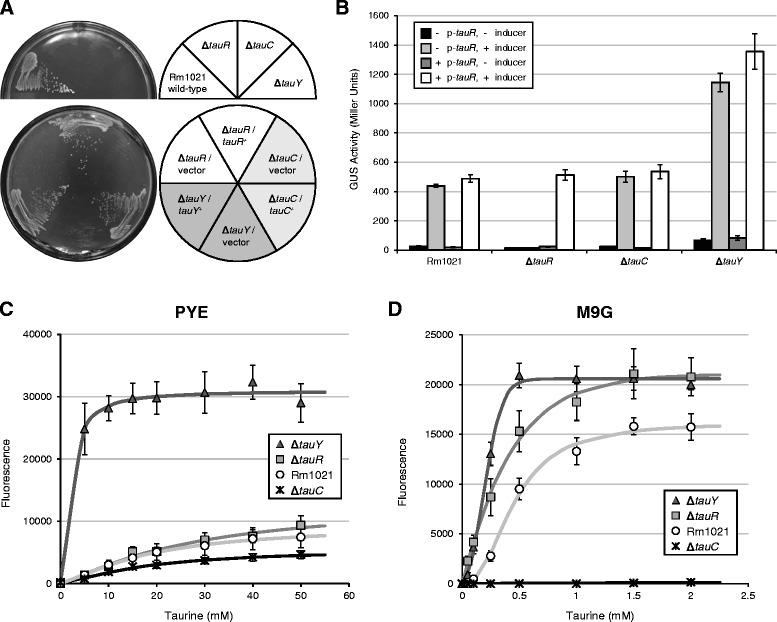
**Effects of representative** ***tau*** **mutations on taurine metabolism and P**_***tauA***_ **expression. (A)** *S. meliloti* Rm1021 wild-type and *tau* mutants were grown on M9 minimal medium supplemented with 100 mM taurine as the sole carbon source. Plate images, captured after 7 days of incubation at 30°C, are shown on the left, while strain genotypes are shown on the right. Strains used were Rm1021, JOE3844, JOE3846, and JOE3848; plasmids used were pJC478, pJC479, pJC538, and pJC539. **(B)** Expression of a plasmid-borne P_*tauA*_-*uidA* reporter was assessed in Rm1021 wild-type and *tau* mutant backgrounds in PYE rich medium. Strains with reporter plasmids containing or lacking the *tauR* gene (+ or - p-*tauR*) were grown in PYE with or without 10 mM taurine (+ or - inducer). Error bars represent standard errors. Strains used were Rm1021, JOE3844, JOE3846, and JOE3848; plasmids used were pJC478 and pJC479. Wild-type strains carrying plasmids without the *uidA* gene (pJC472 or pJC473) exhibited negligible levels of GUS activity (<0.1 Miller units; data not shown). **(C,** **D)** Dose-dependent expression of a plasmid-borne P_*tauA*_-*mCherry* reporter was measured in **(C)** PYE and **(D)** M9G media. Strains were grown for 20 hours in PYE or M9G supplemented with different concentrations of taurine. Wild type carrying the vector (pBBR1MCS-2) was used to determine background fluorescence levels. Error bars represent standard errors of the mean of at least five biological samples. Strains used were Rm1021, JOE3844, JOE3846, and JOE3848.

We first constructed strains with individual in-frame deletions of *tauR*, *tauC*, or *tauY* and tested their ability to utilize taurine as the sole carbon and energy source (Figure 5A). The Rm1021 wild-type strain formed visible colonies on M9 minimal medium supplemented with taurine after five to seven days of incubation at 30°C, whereas all three *tau* mutants failed to exhibit detectable growth. Thus, *tauR*, *tauC*, and *tauY* are each required for taurine dissimilation in *S. meliloti*. Growth was restored when each mutant was complemented with the appropriate gene on a plasmid, suggesting that the individual deletions did not deter expression of downstream genes; *i.e.*, they were not polar mutations.

To assess the effects of the *tau* deletions, we used two similar plasmids carrying the P_*tauA*_*-uidA* reporter fusion, one with *tauR* also on the plasmid (pJC479), and the other without (pJC478). Wild-type and mutant strains transformed with the plasmids were grown in PYE in the presence or absence of taurine for three hours and then harvested to measure gene expression (Figure 5B). Except for the Δ*tauR* mutant, all strains showed similar patterns of expression with both reporter plasmids: low basal activities in the absence of taurine and high levels of expression when induced. The Δ*tauR* mutant exhibited induction when *tauR* was present on the reporter plasmid but no induction when *tauR* was absent, confirming that TauR is the transcriptional activator for the P_*tauA*_ promoter, as previously shown [28]. Behavior of the Δ*tauC* mutant was indistinguishable from that of the wild type, suggesting that the ABC transporter is not required for taurine to enter the cell in complex medium. Finally, whether induced or uninduced, the Δ*tauY* mutant displayed the highest levels of reporter expression compared to other strains grown under similar conditions. These results are consistent with an increased accumulation of taurine, and thus enhanced expression, in the Δ*tauY* mutant due to its inability to degrade the sulfonate compound. Higher basal expression may be attributed to low levels of taurine in the PYE complex medium itself.

Somewhat unexpectedly, deletion of *tauC*, which encodes a component of the putative taurine transporter, did not affect P_*tauA*_ expression in PYE medium; because *tauC* is required for taurine utilization (Figure 5A), we decided to assess the effects of the *tau* mutations over a range of inducer concentrations in both complex and defined media. To facilitate measurement of promoter activity, we generated a plasmid bearing *tauR* as well as a P_*tauA*_*-mCherry* reporter fusion. Wild-type and deletion strains carrying the plasmid were grown for 20 hours with different concentrations of taurine in PYE or M9G prior to measurement of mCherry fluorescence. For all strains in PYE rich medium (Figure 5C), an increase in fluorescence directly correlated with an increase in taurine concentration. As expected, since the *tauR* mutation is complemented by a copy of *tauR* on the plasmid, the wild-type Rm1021 and Δ*tauR* strains behaved similarly. Moreover, in agreement with the GUS assay results (Figure 5B), the Δ*tauY* mutant exhibited much higher levels of expression compared to the other strains, reaching saturation at relatively low levels of inducer (10–20 mM taurine). The Δ*tauC* mutant displayed slightly lower expression of P_*tauA*_*-mCherry* compared to the wild type, with the difference becoming more noticeable at higher concentrations of taurine. This subtle difference in expression suggests that while TauC and its associated ABC-type transporter are not absolutely required for taurine import in complex medium, they may enhance the process.

In contrast, TauC is required in M9G minimal medium for import of taurine to induce P_*tauA*_ expression. Only basal levels of fluorescence were observed with the Δ*tauC* mutant, regardless of taurine concentration, compared to the wild type and other *tau* mutants, where fluorescence correlated with increasing taurine concentration (Figure 5D). Similar to results obtained with the GUS reporter (Figure 4B), wild-type Rm1021 required much lower levels of taurine to achieve full induction in M9G compared to PYE. The Δ*tauY* mutant again exhibited the highest levels of expression. Intriguingly, in M9G medium the Δ*tauR* mutant showed consistently higher expression relative to wild-type Rm1021 and reached levels similar to that of the Δ*tauY* mutant at higher taurine concentrations. We do not have a clear explanation for this observation. The Rm1021 strain actually carries two copies of *tauR* (one genomic, one plasmid-borne) and should activate expression more readily than the Δ*tauR* mutant, which only has one copy of the transcriptional regulator gene on the plasmid. One possibility is that genomic deletion of *tauR* influences expression of surrounding genes, such as reducing the levels of taurine dehydrogenase, and thus affects expression from the reporter plasmid.

Taken together, our results indicate that *tauR* encodes a transcriptional activator necessary for expression from the P_*tauA*_ promoter. TauY is involved in the degradation of taurine, most likely by forming part of the taurine dehydrogenase. The ABC transporter that contains TauC is required to import taurine in minimal, but not complex, medium. We speculate that the ABC transporter encoded by SMc02829-2832 serves as an alternative taurine uptake pathway in complex medium, because SMc02832 expression is induced by taurine, valine, isoleucine, and leucine [25].

### Taurine-dependent expression in *C. crescentus* and *Z. mobilis*

**Figure 6:**
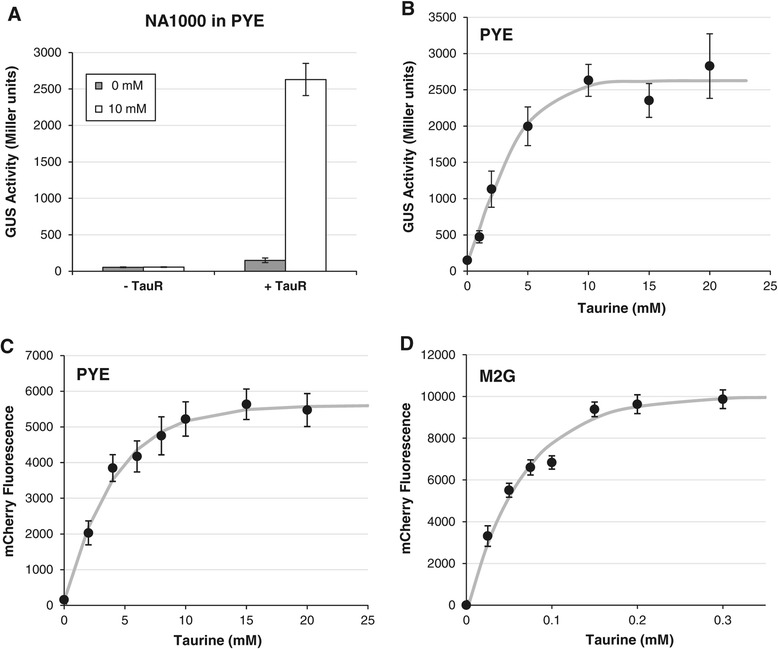
**Expression of plasmid-borne transcriptional fusions to P**_***tauA***_ **in** ***C. crescentus*** **NA1000. (A)** NA1000 carrying P_*tauA*_-*uidA* reporter plasmids, without or with *tauR* (pJC478 or pJC479, respectively), were grown for two hours in PYE rich medium without or with 10 mM taurine and assayed for GUS activity. **(B)** NA1000 carrying pJC479 was induced for two hours with increasing concentrations of taurine in PYE. Wild-type strains carrying plasmids without the *uidA* gene (pJC472 or pJC473) exhibited negligible levels of GUS activity (<0.1 Miller units; data not shown). **(C,** **D)** P_*tauA*_-*mCherry* expression level depends on taurine concentration in **(C)** PYE rich medium or **(D)** M2G defined medium. NA1000 carrying pJC503 was induced for four hours with increasing concentrations of taurine and assayed for fluorescence. Fluorescence values were adjusted by subtracting the background fluorescence of a strain carrying the vector pRVMCS-5. Error bars represent standard errors of the mean of at least six independent measurements.

To explore the usefulness of the P_*tauA*_ promoter in other alpha-proteobacteria, we investigated its dependence on taurine for expression in *C. crescentus*, a model system for studying bacterial cell cycle progression and organelle biogenesis [17]. Wild-type NA1000 carrying reporter plasmids with the P_*tauA*_*-uidA* fusion were harvested for GUS assays after two hours of growth in PYE medium with or without taurine. When the reporter plasmid also contained *tauR* (pJC479), GUS activity was high in the presence of taurine and low in its absence, indicating induction (Figure 6A, +TauR). When the reporter plasmid lacked *tauR* (pJC478), GUS activities remained low regardless of taurine concentration (Figure 6A, −TauR). These results confirmed that TauR is necessary to activate expression from the P_*tauA*_ promoter. Furthermore, *C. crescentus* does not have a functional homolog to substitute for *S. meliloti* TauR.

The same P_*tauA*_*-uidA* reporter strain (with *tauR*) was used to determine the possible range of expression in PYE medium (Figure 6B). GUS activity increased with taurine concentration and reached maximum at approximately 10 mM taurine. Thus, much lower levels of the inducer were needed to saturate the P_*tauA*_ expression system in *C. crescentus* relative to *S. meliloti*. Expression varied approximately 19-fold, from 150 to 2800 Miller units. Similar results were obtained using a reporter plasmid carrying *tauR*-P_*tauA*_*-mCherry*: fluorescence levels increased with taurine concentration and approached maximum at 10 mM taurine in PYE (Figure 6C) and 0.15 mM taurine in M2G minimal medium (Figure 6D). Therefore, the P_*tauA*_ promoter should be useful for modulating gene expression in *C. crescentus*.

**Figure 7:**
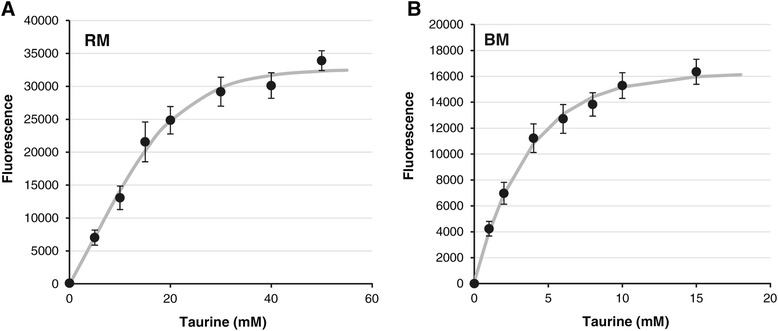
**Dose-dependent expression of a plasmid-borne P**_***tauA***_ **-** ***mCherry*** **reporter in** ***Z. mobilis*** **.** Strains carrying pJC514 were grown for 20 hours in **(A)** RM rich medium or **(B)** BM basal medium supplemented with increasing concentrations of taurine and assayed for mCherry fluorescence. Wild-type ZM4 carrying the vector (pBBR1MCS-2) was used to determine and subtract the background fluorescence levels. Error bars represent standard errors of the mean of at least four independent biological samples.

We also examined P_*tauA*_ expression in a more distantly related alpha-proteobacterium, *Z. mobilis*, which possesses notable ethanol production properties of particular interest for biofuel synthesis [44]. Wild-type ZM4 carrying *tauR*-P_*tauA*_*-mCherry* on a plasmid were grown in RM complex or BM defined media with increasing concentrations of taurine. Fluorescence measurements yielded trends similar to those observed in *S. meliloti* and *C. crescentus*. First, increases in fluorescence correlated with increases in taurine concentrations in both RM (Figure 7A) and BM (Figure 7B) media. Second, the expression system approached maximal induction at lower taurine concentrations in defined medium (15 mM taurine in BM) compared to complex medium (50 mM taurine in RM). Thus, the P_*tauA*_ promoter can regulate gene expression in *Z. mobilis*. Moreover, because we observed taurine-dependent expression in both *C. crescentus* and *Z. mobilis*, which belong to different phylogenetic orders (*Caulobacterales* and *Sphingomonadales*, respectively), the P_*tauA*_ promoter from *S. meliloti* (of the order *Rhizobiales*) may serve as a convenient genetic tool in divergent members of the class *Alphaproteobacteria* [1,2].

### Depletion of essential proteins in *S. meliloti*

**Figure 8:**
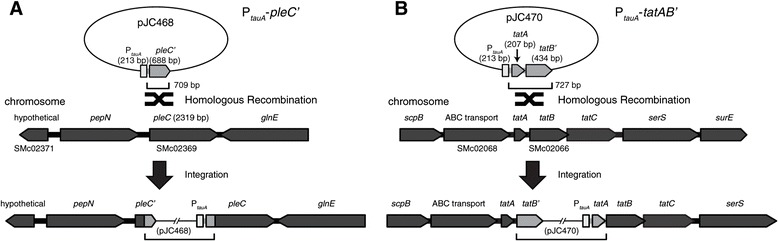
**Schematics depicting integration of plasmids into the** ***S. meliloti*** **chromosome to generate depletion strains.** Integration of **(A)** pJC468 or **(B)** pJC470 placed the only intact copy of *pleC* or *tatB*, respectively, under control of the P_*tauA*_ promoter. The sizes of the regions that enabled homologous recombination are shown below each plasmid.

To demonstrate the utility of the P_*tauA*_ promoter, we constructed showcase *S. meliloti* strains that allowed depletion of cellular factors essential for viability. Essential genes were placed under the control of the P_*tauA*_ promoter, which enabled expression in the presence of taurine and shut off expression in its absence. The first example we chose was *pleC*, encoding a conserved histidine kinase required for cell division in *S. meliloti* [45]. The 5’ portion of *pleC* was transcriptionally fused to P_*tauA*_ on a suicide plasmid, which was subsequently integrated into the native *pleC* locus via homologous recombination, such that the only intact copy of *pleC* is regulated by P_*tauA*_ (Figure 8A). The resultant strain (JOE3601) has already been shown to require taurine for viability [45] and is included here for comparison. Another depletion strain (JOE3608), in which the only copy of *pleC* is under the control of P_*tauA*_ on a plasmid, also exhibited a similar dependence on taurine for growth [45].

The second example we chose was *tatABC*, three contiguous genes that encode essential components of the twin arginine transport system in *S. meliloti* [46]. We took an approach similar to that for *pleC*. Because *tatA* is relatively small in size, and we wanted >500 bp of homology for efficient recombination, both *tatA* and the 5’ portion of *tatB* were fused to P_*tauA*_ on the suicide plasmid. The resultant depletion strain has *tatABC* under control of P_*tauA*_, but also an intact copy of *tatA* under control of its native promoter (Figure 8B). Presumably TatB and TatC are depleted in this strain when expression is blocked in the absence of taurine.

**Figure 9:**
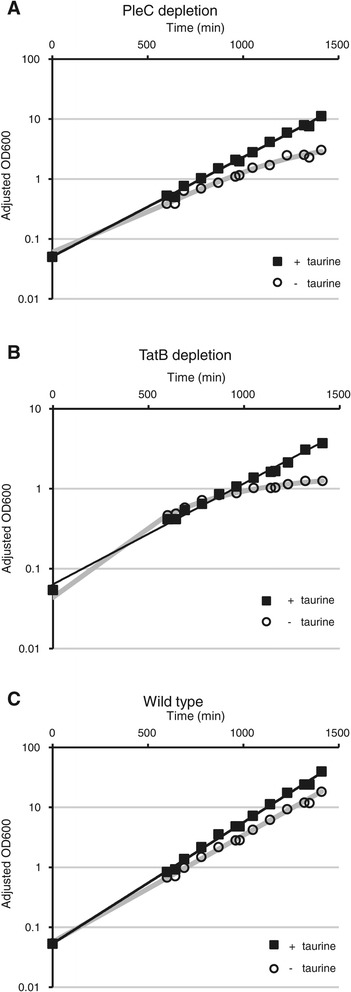
**Representative growth curves showing depletion of PleC and TatB. (A)** PleC depletion (JOE3601), **(B)** TatB depletion (JOE3604), and **(C)** wild-type Rm1021 strains were grown in PYE rich medium without or with 100 mM taurine. Absorbance at 600 nm (OD600) was used to monitor the growth of cultures, and measurements were adjusted accordingly when cultures were diluted to maintain growth in log phase.

The PleC and TatB depletion strains grew normally, similar to wild-type, in the presence of taurine in PYE rich medium but stopped duplicating when grown in medium lacking taurine (Figure 9). The PleC depletion strain began slowing its growth 600 minutes (10 hours) after shifting into medium without taurine, and halted approximately 1320 minutes (22 hours) after the shift. The TatB depletion strain showed slower growth 960 minutes (16 hours) after medium shift and stopped growing also after approximately 1320 minutes. In contrast, wild-type Rm1021 displayed typical doubling regardless of taurine availability, except that the presence of taurine seemed to stimulate slightly more robust growth, most likely due to its use as a nutritional source. These results indicate that the P_*tauA*_ promoter can exert tight control over the expression of essential genes and facilitate studies of physiological consequences when vital cellular components are depleted.

## Conclusion

Together with previously published results [28,29], our analysis demonstrates that the P_*tauA*_ promoter of *S. meliloti* Rm1021, when paired with its cognate transcriptional activator TauR, can provide robust tuning of gene expression in diverse model organisms of the class *Alphaproteobacteria*, including members of *Rhizobiales* (*R. leguminosarum* Rlv3841, *R. etli* CE3, and *S. fredii* NGR234), *Rhodobacterales* (*R. sphaeroides* WS8N), *Sphingomonadales* (*Z. mobilis* ZM4), and *Caulobacterales* (*C. crescentus* NA1000). Import of taurine does not appear to pose a challenge, despite the absence of known or obvious transporters for the inducer in many of the organisms tested. P_*tauA*_ and *tauR*, as well as other promoter regions described above, can be readily amplified from plasmids reported here and elsewhere [28,29] and cloned into other vectors that are more suitable for the end user. Therefore, this study adds to the repertoire of tools available for analyzing genetic models of *Alphaproteobacteria*. Because taurine has been shown to activate expression of genes other than those in the *tau* region, such as *rpoE1* and *rpoE4*, in *S. meliloti* [47], the addition of taurine as an inducer may cause ancillary and potentially undesirable effects, particularly in untested organisms. Thus, appropriate control strains, such as one harboring an empty vector, should be included for comparison when examining the physiological consequences of inducing a gene of interest under the control of P_*tauA*_.

In its native host, P_*tauA*_ permits depletion of essential proteins and tight regulation of potentially toxic genes, especially if combined with careful manipulation of translational efficiency; specifically, Harrison *et al*. [29] was able to reduce the level of expression from P_*tauA*_ by modifying sequences adjacent to the ribosome binding site such that they form RNA secondary structures. Overexpression of the gene under P_*tauA*_ control can be conducted in the Δ*tauY* background, in which inducer level remains high due to the inability to degrade taurine. Considering the ranges of expression observed, similar levels of control in other host organisms should be achievable with appropriate adjustments, including alterations of copy number via placement in the genome or on plasmids. The possibility of using the same construct to control expression in different alpha-proteobacteria can expedite examination of gene function across species: for example, we can use the same plasmid to determine whether an essential gene from one organism can complement null mutations in its orthologs. Although P_*tauA*_ currently does not appear regulatable outside of the alpha-proteobacteria group, for instance in *E. coli* or *Pseudomonas aeruginosa* [28], further investigation and engineering may broaden the range of its functionality.

## Methods

### Bacterial strains, growth conditions, and genetic techniques

Strains and plasmids used in this study are listed in Tables 1 and 2. *S. meliloti* strains were grown at 30°C in LB (1% tryptone, 0.5% yeast extract, 1% NaCl) [48], PYE (0.2% peptone, 0.1% yeast extract, 1 mM MgSO_4_; 0.5 mM CaCl_2_ added only for liquid medium) [49,50], or M9 minimal medium [51], with antibiotics when appropriate: streptomycin (250 μg ml^−1^), neomycin (50 or 100 μg ml^−1^), gentamicin (20 μg ml^−1^), and oxytetracycline (0.5 or 1 μg ml^−1^). M9G contained 0.2% D-glucose as carbon source, M9 + CAA contained 0.2% casamino acids, and, for counter-selection against *sacB*, PYE contained 3% sucrose. *C. crescentus* NA1000 and its derivatives were grown at 30°C in PYE or M2G media [49], with nalidixic acid (20 μg ml^−1^) and oxytetracycline (1 μg ml^−1^) when appropriate. (M2 minimal medium was supplemented with 0.2% glucose to make M2G.) *Z. mobilis* ZM4 and its derivatives were grown at 30°C in RM or BM media [52], with nalidixic acid (20 μg ml^−1^) and kanamycin (200 μg ml^−1^) when appropriate. The original recipe for BM medium was modified by using yeast nitrogen base (YNB) without amino acids and ammonium sulfate (Difco) as the source of vitamins and trace elements. To make BM, we first dissolved and autoclaved 0.1 g K_2_HPO_4_, 0.1 g (NH_4_)_2_SO_4_, 40 mg NaCl, and 2 g glucose in 90 mL water; after the solution cooled, we added the following filter-sterilized solutions: 0.9 mL 10 mM FeSO_4_, 1 mL 10 mM Na_2_MoO_4_, and 10 mL 10X YNB (1.7 g YNB without amino acids and ammonium sulfate dissolved in 100 mL water). *E. coli* strains were grown at 37°C in LB, supplemented with gentamicin (15–20 μg ml^−1^), kanamycin (30–50 μg ml^−1^), or oxytetracycline (12 μg ml^−1^) when appropriate. All solid media contained 1.5% agar. Stock concentrations of casamino acids and various sugars were each 20% (w/v), while that of taurine was 0.5 M.

**Table 1:** **Strains used in this study**

**Strain**	**Relevant genetic markers, features, and/or description**	**Construction, source, or reference**
DH5α	*Escherichia coli* cloning strain: *F* ^*−*^ *φ80lacZΔM15* Δ(*lacZYA-argF*)U169 *endA1 recA1 hsd*R17 (rk-, mk+) *deoR thi-1 supE44 gyrA96 relA1*	Invitrogen
DH10B	*Escherichia coli* cloning strain: *F* ^*−*^ *mcrA* Δ*(mrr-hsdRMS-mcrBC) φ80lacZΔM15* Δ*lacX74 endA1 recA1 deoR* Δ*(ara, leu)7697 araD139 galU galK nupG rpsL*	Invitrogen
MT616	MT607/pRK600; *Escherichia coli* helper strain for mobilizing RK2/RP4-derived plasmids	[53]
NA1000	*Caulobacter crescentus syn-1000*; previously called CB15N, a synchronizable derivative of wild-type CB15	[54]
ZM4	*Zymomonas mobilis* wild-type strain, previously called CP4	[55]
Rm1021	*Sinorhizobium meliloti* SU47 derivative, Sm^R^ (progenitor of strains listed below)	[56]
JOE3273	SMb20895::pJC445 (P_*araA*_ *-uidA*)	Mate pJC445 into Rm1021, select for Nm^R^
JOE3275	SMb21526::pJC446 (P_*tauA*_ *-uidA*)	Mate pJC446 into Rm1021, select for Nm^R^
JOE3277	SMc02323::pJC447 (P_*rhaR*_ *-uidA*)	Mate pJC447 into Rm1021, select for Nm^R^
JOE3334	SMb21648::pJC455 (P_*melA*_ *-uidA*)	Mate pJC455 into Rm1021, select for Nm^R^
JOE3601	SMc02369::pJC468 (P_*tauA*_-*pleC*)	Mate pJC468 into Rm1021, select for Nm^R^
JOE3604	SMc02066::pJC470 (P_*tauA*_-*tatAB’*)	Mate pJC470 into Rm1021, select for Nm^R^
JOE3844	Δ*tauR* (ΔSMb21525)	Allelic replacement using pJC507
JOE3846	Δ*tauC* (ΔSMb21528)	Allelic replacement using pJC508
JOE3848	Δ*tauY* (ΔSMb21529)	Allelic replacement using pJC509

**Table 2:** **Plasmids used in this study**

**Plasmid**	**Relevant genetic markers, features, and/or description**	**Construction, source, or reference**
pBBR1MCS-2	Broad-host-range vector, Km^R^	[57]
pCM130	RK2-derived broad-host-range vector with *E. coli rrnB* terminator preceding polylinker, Tc^R^	[58]
pJQ200sk	Counter-selectable vector for allelic replacement, *sacB*, Gm^R^	[59]
pRVMCS-5	RK2-derived broad-host-range vector with *C. crescentus* P_*van*_ promoter, Tc^R^	[14]
pVCHYN-5	Narrow-host-range vector with *C. crescentus* P_*van*_ promoter and *mCherry*, Tc^R^	[14]
pVO155	pUC119-derived suicide vector with *uidA* cassette, Nm^R^/Km^R^ Ap^R^	[60]
pJC445	pVO155-P_*araA*_, for fusing *uidA* to sequence upstream of *araA*	This study
pJC446	pVO155-P_*tauA*_, for fusing *uidA* to sequence upstream of *tauA*	This study
pJC447	pVO155-P_*rhaR*_, for fusing *uidA* to sequence upstream of *rhaR*	This study
pJC455	pVO155-P_*melA*_, for fusing *uidA* to sequence upstream of *melA*	This study
pJC468	pVO155-P_*tauA*_-*pleC*’, for integrating into chromosome and placing *pleC* under control of P_*tauA*_	This study
pJC470	pVO155-P_*tauA*_-*tatAB*’, for integrating into chromosome and placing *tatB and tatC* under control of P_*tauA*_	This study
pJC472	pCM130-P_*tauA*_	This study
pJC473	pCM130-*tauR*-P_*tauA*_	This study
pJC474	pRVMCS-5-derived plasmid carrying P_*tauA*_	This study
pJC475	pRVMCS-5-derived plasmid carrying *tauR*-P_*tauA*_	This study
pJC478	pCM130-P_*tauA*_-*uidA*	This study
pJC479	pCM130-*tauR*-P_*tauA*_-*uidA*	This study
pJC503	pRVMCS-5-derived plasmid carrying *tauR*-P_*tauA*_-*mCherry*	This study
pJC507	pJQ200sk-Δ*tauR*	This study
pJC508	pJQ200sk-Δ*tauC*	This study
pJC509	pJQ200sk-Δ*tauY*	This study
pJC514	pBBR1MCS-2-*tauR*-P_*tauA*_-*mCherry*	This study
pJC538	pCM130-P_*tauA*_-*tauC*	This study
pJC539	pCM130-P_*tauA*_-*tauY*	This study

Mobilization of plasmids from *E. coli* to *S. meliloti* , *C. crescentus*, or *Z. mobilis* was accomplished by triparental mating, with the help of strain MT616, carrying pRK600 [53]; nalidixic acid or streptomycin was used to select against *E. coli* donor and helper strains. Electroporation of plasmid DNA into *C. crescentus* was achieved using cuvettes with 1-mm gaps and a Bio-Rad Gene Pulser II with the following settings: 400 Ω, 25 μF, and 1.5 kV [50]. Deletions in the *S. meliloti* genome were generated by homologous recombination and allelic replacement, using a two-step selection procedure with the vector pJQ200sk [59,61]. Plasmid integrations and genomic deletions were screened and confirmed by PCR. Construction of depletion strains required the presence of 100 mM taurine in the medium while selecting for transconjugants.

### Molecular cloning

Standard techniques were used for cloning and analysis of DNA, PCR, and transformation into *E. coli* [51,62]. Plasmids and DNA fragments were isolated using commercial kits from Qiagen. KOD polymerase (Novagen) or MangoMix (Bioline) was used for high- or low-fidelity PCR amplification, respectively, while other enzymes used to manipulate DNA came from New England BioLabs. Elim Biopharmaceuticals provided oligonucleotide synthesis and DNA sequencing services. Details of plasmid construction are described in Additional file 1. Oligonucleotide sequences are provided in Additional file 2.

### Beta-glucuronidase (GUS) reporter assays

We monitored expression of the *uidA* reporter, under the control of various promoters, by assessing beta-glucuronidase activities as previously described [63], except lysozyme was omitted and 400 μL 1 M sodium carbonate was added to stop each reaction. Enzymatic activity in each biological sample was measured in duplicates or triplicates, and measurements from at least three independent biological samples were averaged.

For comparison of different promoters, *S. meliloti* strains were grown in PYE, LB, or M9 liquid media overnight, diluted to the desired optical density, and induced for three hours, to mid-log phase, with 0.2% (13.3 mM) L-arabinose, 0.063% (5 mM) taurine, 0.2% (11.0 mM) L-rhamnose monohydrate, 0.2% (5.6 mM) D-melibiose monohydrate, or 0.2% (3.4 mM) D-raffinose pentahydrate. For induction in M9, strains were grown in M9 supplemented with 0.2% glucose (M9G), washed in M9 buffer without carbon source, and diluted into M9G or M9 plus inducer as the sole carbon source. For induction in M9 supplemented with 0.2% casamino acids (M9 + CAA), strains were either first grown in M9 + CAA without glucose and then diluted into the same medium with or without inducer; or they were first grown in M9 + CAA supplemented with 0.2% glucose, washed with M9 + CAA, and diluted into M9 + CAA supplemented with either glucose or the inducer.

For comparison of melibiose and raffinose as inducers of P_*melA*_, strain JOE3334 was grown in PYE in the presence or absence of the inducer for three hours to mid-log phase, for 20 hours to stationary phase, or for 12 hours to mid-log or stationary phase, by diluting the starter PYE cultures appropriately.

For induction with different concentrations of taurine, *S. meliloti* strains were grown for three hours in PYE or M9G to mid-log phase. For induction with taurine concentrations above 10 mM in PYE, cultures were adjusted to the desired optical density by mixing appropriate volumes of the starter culture, 0.5 M taurine, water, and double-strength PYE (2X PYE), to prevent excessive dilution of nutrients in the medium. Similar procedures were used to measure GUS activity in *C. crescentus*, except the strains were induced for two hours.

### Measurement of mCherry fluorescence

To monitor mCherry expression in *S. meliloti*, we cultivated strains harboring pBBR1MCS-2 or pJC514 overnight in PYE or M9G supplemented with neomycin (5 μg ml^−1^) and then diluted the cultures in media containing different concentrations of taurine, to a final volume of 0.8 mL in each well of a 48-well microtiter plate. The plates were shaken for 20 hours, and 150 μL of each culture was transferred to a 96-well optic plate in duplicates for absorbance (535 nm) and fluorescence (550 nm excitation, 610 nm emission) measurements with a Tecan Spectrafluor Plus reader. Expression in *Z. mobilis* was monitored in a similar fashion, except the strains were grown in RM or BM supplemented with kanamycin (100 μg ml^−1^). For *C. crescentus*, NA1000 carrying pJC503 or pRVMCS-5 was induced in PYE or M2G (M2 plus 0.2% glucose) containing oxytetracycline (0.5 μg ml^−1^) and different concentrations of taurine for four hours. Absorbance readings confirmed similar levels of growth for all samples of the same species. Fluorescence readings were adjusted by subtracting the background fluorescence of strains carrying only the vector (pBBR1MCS-2 or pRVMCS-5). Average background fluorescence readings (in arbitrary units) were approximately 10000 and 21000 for *S. meliloti* in PYE and M9G, 23000 and 30000 for *C. crescentus* in PYE and M2G, and 14000 and 22000 for *Z. mobilis* in RM and BM. Measurements of at least four biological samples, grown on separate days, were averaged following appropriate subtraction of background readings.

## Additional files

Additional file 1:
**Plasmid construction.** Construction of each plasmid is described in detail in this file. Additional file 2:
**Primers used.** This file contains a table of primers used in this study and their sequences.
