# Supplementary material for: Analysis of a taurine-dependent promoter in Sinorhizobium meliloti that offers tight modulation of gene expression
Source: BMC Microbiol. 2014 Nov 25;14:295. doi: 10.1186/s12866-014-0295-2 (PMC4254191; doi:10.1186/s12866-014-0295-2)
Supplement: Additional file 1: — Plasmid construction. Construction of each plasmid is described in detail in this file. [file 12866_2014_295_MOESM1_ESM.pdf]

Plasmid construction

Details of how each plasmid was constructed for this study are described below.

pJC445 was constructed by amplifying a 603-bp region that includes the 5' portion of SMb20895 and its upstream sequence, with primers araA -573F SpeI and araA 29R XhoI, and inserting the resultant fragment into pVO155 after digestion with SpeI and XhoI.

pJC446 was constructed by amplifying a 613-bp region that includes the 5' portion of SMb21526 and its upstream sequence, with primers tauA -577F SpeI and tauA 35R XhoI, and inserting the resultant fragment into pVO155 after digestion with SpeI and XhoI.

pJC447 was constructed by amplifying a 661-bp region that includes the 5' portion of SMc02323 and its upstream sequence, with primers rhaR -616F SpeI and rhaR 44R XhoI, and inserting the resultant fragment into pVO155 after digestion with SpeI and XhoI.

pJC455 was constructed by amplifying a 662-bp region that includes the 5' portion of SMb21648 and its upstream sequence, with primers melA -536F SpeI and 125R XhoI, and inserting the resultant fragment into pVO155 after digestion with SpeI and XhoI.

pJC468 was constructed by amplifying P_{tauA} with primers tauR 1423F SpeI and tauA 3R BamHI and digesting the 231-bp fragment with SpeI and BamHI, amplifying the 5' portion of *pleC* with primers pleC -21F BamHI and pleC 687R XhoI and digesting the 727-bp fragment with BamHI and XhoI, and inserting the resultant fragments into pVO155 digested with SpeI and XhoI.

pJC470 was constructed by amplifying P_{tauA} with primers tauR 1423F SpeI and tauA 3R BamHI and digesting the 231-bp fragment with SpeI and BamHI, amplifying *tatA* and the 5' portion of *tatB* with primers tatA -13F BamHI and tatB 433R XhoI and digesting the 745-bp fragment with BamHI and XhoI, and inserting the resultant fragments into pVO155 digested with SpeI and XhoI.

pJC472 was constructed by amplifying P_{tauA} with primers tauR 54R EcoRI and tauA -3R NB and inserting the resultant 231-bp fragment into pCM130 after digestion with EcoRI and BamHI.

pJC473 was constructed by amplifying *tauR*-P_{tauA} with primers tauR +19R EcoRI and tauA -3R NB and inserting the resultant 1671-bp fragment into pCM130 after digestion with EcoRI and BamHI.

pJC474 was constructed by amplifying the region upstream of *tauA* with primers tauR +19R Hind3 and tauA -4R NE, digesting the product with HindIII and NdeI, and inserting the 302-bp fragment containing P_{tauA} into the same sites of pRVMCS-5.

pJC475 was constructed by amplifying *tauR*-P_{tauA} with primers tauR +19R Hind3 and tauA -4R NE, digesting the product with HindIII, and inserting the 1351-bp fragment into the HindIII site of pJC474.

pJC478 was constructed by inserting an NcoI-NheI fragment containing *uidA* from pVO155 into pJC472 digested with NcoI and XbaI.

pJC479 was constructed by inserting an NcoI-NheI fragment containing *uidA* from pVO155 into pJC473 digested with NcoI and XbaI.

pJC503 was constructed by inserting an NdeI-KpnI fragment containing *mCherry* from pVCHYN-5 into the same restriction sites of pJC475.

pJC507 was constructed by amplifying the region upstream of *tauR* with primers tauR -563F SphI and tauR 14R EcoRI and digesting it with SphI and EcoRI; amplifying the region downstream of *tauR* with primers tauR 1431F EcoRI and tauR +529R SpeI and digesting it with EcoRI and SpeI; and inserting the two resultant fragments into pJQ200sk digested with SphI and SpeI.

pJC508 was constructed by amplifying the region upstream of *tauC* with primers tauC -553F SpeI and tauC 17R EcoRI and digesting it with SpeI and EcoRI; amplifying the region downstream of *tauC* with primers tauC 804F EcoRI and tauC +544R SphI and digesting it with EcoRI and SphI; and inserting the two resultant fragments into pJQ200sk digested with SphI and SpeI.

pJC509 was constructed by amplifying the region upstream of *tauY* with primers tauY -549F SpeI and tauY 17R EcoRI and digesting it with SpeI and EcoRI; amplifying the region downstream of *tauY* with primers tauY 918F EcoRI and tauY +106R SphI and digesting it with EcoRI and SphI; and inserting the two resultant fragments into pJQ200sk digested with SphI and SpeI.

pJC514 was constructed by inserting a 2650-bp XbaI fragment containing *tauR*-P_{tauA}-*mCherry* from pJC503 into the XbaI site of pBBR1MCS-2.

pJC538 was constructed by amplifying *tauC* with primers tauC -1F XbaI and tauC +4R SacI, digesting the product with SacI and XbaI, and inserting the 843-bp fragment into the same sites of pJC472.

pJC539 was constructed by amplifying *tauY* with primers tauY -13F XbaI and tauY +7R SacI, digesting the product with SacI and XbaI, and inserting the 1431-bp fragment into the same sites of pJC472.
