# Supplementary material for: Analysis of a taurine-dependent promoter in Sinorhizobium meliloti that offers tight modulation of gene expression
Source: BMC Microbiol. 2014 Nov 25;14:295. doi: 10.1186/s12866-014-0295-2 (PMC4254191; doi:10.1186/s12866-014-0295-2)
Supplement: Additional file 2: — Primers used. This file contains a table of primers used in this study and their sequences. [file 12866_2014_295_MOESM2_ESM.pdf]

Primers used in this study

Primer	Sequence	Description
araA -573F SpeI	GCAACTAGTCGCAAtTCGAGGAAGATGGT	amplify region upstream of SMb20895; eliminate XhoI site
araA 29R XhoI	GAACTCGAGAGCCGCGAGAAGCGAAGTAA	amplify region upstream of SMb20895
tauA -577F SpeI	CCAACTAGTGGTCCATCTGACCGTATAGG	amplify region upstream of SMb21526
tauA 35R XhoI	AGCCTCGAGTCCGGACAAGTGCTTGAAAC	amplify region upstream of SMb21526
rhaR -616F SpeI	CGGACTAGTACATGCGGCTTCGGAACATA	amplify region upstream of SMc02323
rhaR 44R XhoI	ATTCTCGAGCTGCACCGCCGACAGGATGAT	amplify region upstream of SMc02323
melA -536F SpeI	CTCACTAGTTCCTCGCCGTTCATGGAGAT	amplify region upstream of SMb21648
melA 125R XhoI	GACCTCGAGCTCGCTCAGATCCGTCAAC	amplify region upstream of SMb21648
tauR 1423F SpeI	AACACTAGTTGGCTGGCCCGGTCAAGGAA	amplify region upstream of SMb21526
tauA 3R BamHI	GGCGGATCCTCATGTCGTTACCCTCTTTG	amplify region upstream of SMb21526
pleC -21F BamHI	GCGGGATCCGAGGACGACAAATTGGATAA	amplify 5' region of SMc02369 for integration
pleC 687R XhoI	AGTCTCGAGCCGAAACACCGGCAAACAGC	amplify 5' region of SMc02369 for integration
tatA -13F BamHI	TCCGGATCCAGGGAGTAAGTGGATGGGTT	amplify *tatAB*' (SMc02067, SMc02066) for integration
tatB 433R XhoI	TGTCTCGAGTGCCTCGATGCCGAGCTCACC	amplify *tatAB*' (SMc02067, SMc02066) for integration
tauR 54R EcoRI	GCTGAATTCTTTGGCTGGCCCGGTCAAGG	amplify region upstream of SMb21526
tauA -3R NB	AGCGGATCCATGGCGTTACCCTCTTTGGTTATGTC	amplify region upstream of SMb21526
tauR +19R EcoRI	TCCGAATTCACCCTCTGCAAGGCTCATTC	amplify SMb21525
tauA -4R NE	AGGGAATTCATATGGTTACCCTCTTTGGTTATGTC	amplify region upstream of SMb21526
tauR + 19R Hind3	TCCAAGCTTACCCTCTGCAAGGCTCATTC	amplify SMb21525
tauR -563F SphI	ACGGCATGCTTCTTTCCCTTCAGGTCTTC	amplify region 5' of *tauR* (SMb21525)
tauR 14R EcoRI	TTGGAATTCGGTCGAGCCCGCCATTGTTC	amplify region 5' of *tauR* (SMb21525)
tauR 1431F EcoRI	TTAGAATTCGACGGCGTGGCCCTGACTGC	amplify region 3' of *tauR* (SMb21525)
tauR +529R SpeI	GGAACTAGTGGACGCGGTGCCGATTCTGC	amplify region 3' of *tauR* (SMb21525)
tauC -553F SpeI	GTCACTAGTCAGAAGCACGCGCTTCTCCC	amplify region 5' of *tauC* (SMb21528)
tauC 17R EcoRI	CTTGAATTCTGCGACACCCTCAAGCATGG	amplify region 5' of *tauC* (SMb21528)
tauC 804F EcoRI	CGCGAATTCATTCCCTGGAAGGGCAAAGTG	amplify region 3' of *tauC* (SMb21528)
tauC +544R SphI	ATGGCATGCGCCAGTTGGTGTTCCATGTC	amplify region 3' of *tauC* (SMb21528)
tauY -549F SpeI	GCTACTAGTCCCTGGAAGGGCAAAGTGTG	amplify region 5' of *tauY* (SMb21529)
tauY 17R EcoRI	GAGGAATTCGGCCAGTTGGTGTTCCATGTC	amplify region 5' of *tauY* (SMb21529)
tauY 918F EcoRI	GCCGAATTCAACTTCAAGACGCGGATCCC	amplify region 3' of *tauY* (SMb21529)
tauY +106R SphI	AACGCATGCTCGATGCCGTGCATCTG	amplify region 3' of *tauY* (SMb21529)
tauC -1F XbaI	GCATCTAGACATGCTTGAGGGTGTCGCAAAGAG	amplify *tauC* (SMb21528)
tauC +4R SacI	TGAGAGCTCGCCGTCACACTTTGCCCTTC	amplify *tauC* (SMb21528)
tauY -13F XbaI	GCATCTAGAGCAGGATCACGACATGGAAC	amplify *tauY* (SMb21529)
tauY +7R SacI	AATGAGCTCCGGGCGGCTAAAGAATTTCG	amplify *tauY* (SMb21529)
